# Characterization of *CaPEX8* in Peroxisome Biogenesis and Pathogenicity of *Colletotrichum aenigma*

**DOI:** 10.3390/jof12040241

**Published:** 2026-03-26

**Authors:** Yan-Xi Lin, Ying-Ying Cai, Shen-Dan Yu, Jing Wang, Xin-He Wang, Zhong-Na Hao, Zhen Zhang, Hai-Ping Qiu, Rong-Yao Chai, Yan-Li Wang, Qian-Sheng Liao, Jiao-Yu Wang

**Affiliations:** 1 College of Life Sciences and Medicine, Zhejiang Sci-Tech University, Hangzhou 310018, China; linyanxi0227@163.com (Y.-X.L.); liaoqs@zstu.edu.cn (Q.-S.L.); 2 State Key Laboratory for Quality and Safety of Agro-Products, Key Laboratory of Agricultural Microbiome (MARA), Key Laboratory of Agricultural Microbiome of Zhejiang Province, Key Laboratory of Biotechnology in Plant Protection of MARA, Zhejiang National Crop Variety Resistance Identification Experiment Station, Institute of Plant Protection and Microbiology, Zhejiang Academy of Agricultural Sciences, Hangzhou 310021, China; caiyy@zaas.ac.cn (Y.-Y.C.);

**Keywords:** *Colletotrichum aenigma*, peroxisome, *PEX8*, pathogenicity

## Abstract

Peroxisomes are ubiquitous organelles that play vital roles in various physiological and biochemical processes, including fatty acid β-oxidation and reactive oxygen species (ROS) metabolism. These organelles have been implicated in the pathogenicity of many plant fungal pathogens. In this study, CaPex8, a homolog of *Saccharomyces cerevisiae* Pex8, was identified and characterized in *Colletotrichum aenigma*. *CaPEX8* was found to localize to peroxisomes, and its deletion impaired the mutant’s ability to utilize fatty acids as a carbon source. Using a green fluorescent protein (GFP) fused to the peroxisomal targeting signal PTS1, the import of peroxisomal matrix proteins was shown to be defective in Δ*Capex8* mutants. Additionally, the mutants exhibited elevated conidiation, increased sensitivity to osmotic stress and oxidative stress, and impaired cell wall integrity. Peroxisome biogenesis was also disrupted in the absence of *CaPEX8*. Taken together, these results demonstrate that CaPex8 is essential for maintaining peroxisomal structure and function, and it significantly influences fungal growth, development, and pathogenicity in *C. aenigma*.

## 1. Introduction

*Colletotrichum aenigma* is a filamentous ascomycete fungus responsible for grape anthracnose, also known as grape late rot [1]. Infection by this pathogen leads to the formation of characteristic dark brown anthracnose lesions on fruits, leaves, and stems of host plants. Infected fruits often exhibit exudation and decay, resulting in substantial yield losses and a significant decline in product quality [1,2]. *C. aenigma* exhibits a broad host range, including grapes, strawberries [3], apples [4], *Actinidia arguta* [5], and other economically significant crops. The extensive host adaptability of this pathogen poses a serious threat to agricultural production, making it imperative to gain in-depth insights into its pathogenic mechanisms.

Peroxisomes are single-membrane-bound organelles, ranging from 0.2 to 1.0 μm in diameter, and are ubiquitous in most eukaryotic cells [6]. These organelles house over 50 enzymes, including oxidases and catalase, and participate in diverse biochemical metabolic processes, earning them the designation as the cell’s “metabolic hub”. Among these, the most critical functions of peroxisomes are lipid metabolism and the breakdown of reactive oxygen species (ROS) [7]. In *S*. *cerevisiae*, peroxisomes serve as the exclusive site for fatty acid β-oxidation, which is crucial for maintaining cellular fatty acid homeostasis [8]. Recent studies in filamentous fungi have demonstrated that peroxisomes synthesize a range of metabolites, and their loss abolishes fatty acid utilization, thereby causing retarded growth and abnormal organelle morphology on a medium where fatty acids serve as the sole carbon source [9,10,11,12].

Peroxisome biogenesis primarily involves matrix protein import and membrane protein assembly. Matrix proteins are synthesized in the cytoplasm and harbor peroxisomal targeting signals (PTSs) that govern their intracellular sorting. The vast majority of soluble matrix proteins rely on the C-terminally located type I peroxisomal targeting signal (PTS1) to enter the organelle matrix, while a few matrix proteins are imported via the N-terminally located type II targeting signal (PTS2) [13]. The metabolic activity of peroxisomes depends on the correct targeting of matrix proteins. In the cytosol, PTS1 is specifically recognized by the receptor protein *PEX5*, which subsequently mediates the translocation of PTS1-carrying matrix proteins across the membrane and their dissociation within the matrix. This import cascade represents the core mechanism underlying the maintenance of peroxisomal metabolic function [14,15].

To maintain cellular homeostasis, peroxisomes adapt to environmental changes by balancing their own biogenesis and degradation [16]. This process relies on a class of peroxins—proteins encoded by *PEX* genes that facilitate peroxisome assembly. To date, 37 peroxisomal proteins have been identified across multiple species, functioning in membrane protein anchoring and matrix protein import [7,17]. A body of evidence indicates that *PEX* genes play a key role in the pathogenicity mechanisms in a wide range of plant-pathogenic fungi. For instance, in *Colletotrichum orbiculare*, deletion of *PEX6* impairs PTS1-mediated matrix protein import, reduces fatty acid utilisation, and attenuates virulence, while *PEX13* is essential for the formation of infection structures. Similarly, in *Magnaporthe oryzae*, loss of *PEX5* and *PEX7* disrupts both PTS1 and PTS2 pathways, leading to metabolic abnormalities and reduced pathogenicity [18], whereas deletion of *PEX19* results in a complete loss of pathogenicity [10]. In *Fusarium graminearum*, the infection process involves *PEX5* and *PEX6*, among other genes [19]. Collectively, these findings underscore the central role of *PEX* genes in regulating diverse physiological processes in fungi, including hyphal growth, sporulation, lipid utilization, ROS degradation, and virulence. Despite the well-established roles of many *PEX* genes in fungal pathogenicity, the function of *PEX8* in *Colletotrichum* species remains largely unexplored.

Pex8 is a fungus-specific peroxin found uniquely to yeast and filamentous fungi. It has been demonstrated that Pex8 harbors both PTS1 and PTS2 signals and localizes to the inner side of the peroxisomal membrane. As a key component of the peroxisomal importomer, Pex8 connects the *PEX13*/*PEX14* docking complex with the *PEX2*/*PEX10*/*PEX12* RING-finger complex, and interacts with the receptor proteins Pex5 and Pex7, thereby assembling these two complexes into a fully functional peroxisomal protein import machinery [20,21]. These findings underscore the pivotal role of Pex8 in coordinating the functions of multiple peroxins. In *S*. *cerevisiae*, deletion of *PEX8* leads to mis-localization of peroxisomal matrix proteins, a reduction in peroxisome number, and an inability of the Δ*pex8* mutant to grow on oleic acid medium [22]. In *Candida albicans*, the Δ*pex8* mutant shows increased sensitivity to miltefosine-induced reactive oxygen species, indicating the role of Pex8 in peroxisome biogenesis and function [23]. In filamentous fungi, Pex8 also plays critical roles: *BcPEX8* is a key gene regulating peroxisome biogenesis, fatty acid β-oxidation, fungal development, and pathogenicity, and its deletion leads to impairment of multiple physiological functions and a significant reduction in virulence in *B. cinerea* [9]; *PEX8* is essential for peroxisomal matrix protein import and meiocyte formation in *Podospora anserina* [24].

Although *PEX8* has been extensively studied in yeast, its functional mechanisms in filamentous fungi remain poorly understood. In particular, it is still unclear whether *PEX8* is involved in peroxisome biogenesis and pathogenicity in *C. aenigma*. In this study, we constructed a *CaPEX8* deletion mutant and the corresponding complemented strain to dissect the roles of *CaPEX8* in vegetative growth, fatty acid utilization, stress response, and virulence. Using subcellular localization, matrix protein import assays, and transmission electron microscopy, we aimed to elucidate the regulatory mechanism of *CaPEX8* in peroxisome biogenesis and pathogenicity in *C. aenigma*.

## 2. Materials and Methods

### 2.1. Fungal Strains, Growth Conditions and Transformation

The wild-type strain of *C. aenigma*, along with all subsequent transformants and mutant strains, was cultured in complete medium (CM) at 28 °C in the dark. For genomic DNA extraction, mycelia from two-day-old liquid CM cultures were harvested and extracted using a fungal DNA extraction kit (OMEGA, Knoxville, TN, USA). All fungal transformants were obtained via Agrobacterium-mediated transformation (*At*MT) as previously described [25]. Corresponding transformants were screened on CM plates supplemented with 250 mg/mL hygromycin B (Roche, Mannheim, Germany) or on defined complex medium (DCM; 0.16% yeast nitrogen base without amino acids, 0.2% asparagine, 0.1% ammonium nitrate, and 1% glucose, pH 6.0 with Na_2_HPO_4_) [26] containing 100 mg/mL chlorimuron ethyl (Sigma, St. Louis, MO, USA).

### 2.2. Bioinformatic Analysis

Amino acid sequence of each gene used in this study was downloaded from the National Center for Biotechnology Information (NCBI). Phylogenetic analyzes were conducted using MEGA 11.0 software with the neighbor-joining algorithm. Protein sequence alignments were performed using ESPript 3.0 [27]. A phylogenetic tree was constructed using the Neighbor-Joining method in MEGA 11.0.

### 2.3. CaPEX8 Knockout and Complementation

The knockout vector for *CaPEX8* was constructed based on an established gene knockout system [28]. Specifically, a 2 kb upstream fragment (UF) and a 2 kb downstream fragment (DF) of *CaPEX8* were amplified from the genomic DNA of *C. aenigma* using the primer pairs CaPex8up-F/CaPex8up-R and CaPex8down-F/CaPex8down-R, respectively. The hygromycin B (*HPH*) resistance cassette was amplified from the *pCB1300* plasmid using primers *HPH*-F/*HPH*-R. All fragments (UF, DF, and *HPH*) were cloned using Phanta Max Ultra-High Fidelity DNA Polymerase (Vazyme, Nanjing, China), and the resulting fragments were ligated into the linearized (HindIII/XbaI-linearized) vector pKO3A [10]. The knockout vector pKO3A-*CaPEX8* was subsequently introduced into the wild-type strain by *At*MT to generate the Δ*Capex8* mutant.

To construct the gene-complementing vector (∆*Capex8*-C), the full-length fragment of the *CaPEX8* gene was amplified from *C. aenigma* DNA with primers CaPex8-F/CaPex8-R and cloned into the XbaI/SalI-digested backbone vectors pKD6-DsRed and pKD5-GFP. Both vectors are equipped with a sulfonylurea resistance gene (*SUR*). Then, using the *At*MT method, we inserted the obtained gene complemented plasmids into ∆*Capex8* mutant. Successful complementation was initially confirmed by fluorescence observation. One of the confirmed transformants, Δ*Capex8*-C, was used in phenotypic analysis. The primers used in this study are listed in Supplementary Table S1.

PCR amplification was performed in a 50 μL reaction volume containing 2 × Phanta Flash Master Mix (25 μL), forward and reverse primers (2 μL of a 10 μM stock solution each), template (1 μL), and ddH_2_O (20 μL). The amplification program was as follows: initial denaturation at 95 °C for 5 min; 35 cycles of denaturation at 95 °C for 30 s, annealing at 58 °C (depending on the specific primers) for 30 s, and extension at 72 °C for 10 s/kb (adjusted according to product length); followed by a final extension at 72 °C for 10 min. The PCR thermal cycler used was an Eppendorf Mastercycler nexus GSX1, and the polymerase used was 2 × Phanta Flash Master Mix (Vazyme, Nanjing, China).

### 2.4. Phenotypic Characterization

For fungal growth and conidiation assays, 5 mm diameter plugs of *C. aenigma* strains were inoculated on CM and incubated at 28 °C in the dark for 4 days. The diameter was measured after 4 days. For conidial yield quantification, conidia were harvested by rinsing with 4 mL of sterile water from strains cultured for the same duration, filtered, and adjusted to a final volume of 2 mL. The conidial concentration was then determined using a hemocytometer.

### 2.5. Generation of Fluorescent Protein Fusion Constructs

To observe the subcellular localization of CaPex8 in *C. aenigma*, the *CaPEX8* sequence was amplified using genomic DNA as a template. Three fusion expression vectors DsRed-CaPex8, GFP-CaPex8, and CaPex8-GFP, were constructed and subsequently introduced into *C. aenigma* via *At*MT, generating the corresponding fluorescent strains: DsRed-CaPex8, GFP-CaPex8, and CaPex8-GFP.

To determine the localization of the PTS1 in wild-type, Δ*Capex8* mutant, and complemented strains, a previously constructed GFP-PTS1 vector was transformed into these backgrounds using *At*MT, resulting in the fluorescent strains Ca-GFP-PTS1, Δ*Capex8*-GFP-PTS1, and Δ*Capex8*-C-GFP-PTS1.

For colocalization analysis, the GFP-PTS1 vector was introduced into the DsRed-CaPex8 strain, yielding the double-labeled strain DsRed-CaPex8-GFP-PTS1. All strains generated in this study were preserved in our laboratory using the filter paper preservation method.

### 2.6. Strain Fatty Acid Utilization Assays

To assess growth on different fatty acid sources, mycelial plugs (3 × 3 mm) were excised from the periphery of 4-day-old colonies of each strain and inoculated onto minimal medium (MM) supplemented with various carbon sources. The media used included MM (with glucose as the sole carbon source) and MM-C (basal medium containing only nitrogen source, vitamins, and trace elements). MM-C was further supplemented with 0.1% oleic acid, 0.1% Tween 80 (a source of the long-chain fatty acid oleate), or 50 mM sodium acetate (a short-chain carbon source). Cultures were incubated at 28 °C in the dark for 4 days. Colony diameters were measured using the crossover method in all assays.

### 2.7. Stress Susceptibility and Reactive Oxygen Species (ROS) Content Assays

To assess vegetative growth under oxidative stress, 3 × 3 mm mycelial plugs of the wild-type, Δ*Capex8* mutant, and Δ*Capex8-C* were cultured on CM supplemented with 0.1% H_2_O_2_, 50 μM Bengal Red, or 5 mM CuSO_4_ at 28 °C in the dark. For cell wall integrity assays, the three strains were grown on CM containing 50 μg/mL Calcofluor White. Osmotic stress sensitivity was assessed by culturing strains on CM supplemented with 1 M NaCl. After a 5-day incubation period, colony diameters were measured using the crossover method in all assays, and the relative growth inhibition rate was calculated. Each assay described above was independently repeated three times.

Intracellular ROS levels in the wild-type and Δ*Capex8* mutant strains were compared using the Fungal Reactive Oxygen Species Assay Kit (ROS Assay Kit)-Red Fluorescence (BestBio, Shanghai, China) according to the manufacturer’s instructions. Briefly, the wild-type and Δ*Capex8* mutant strains were incubated in CM liquid medium with shaking for 48 h. Mycelia (50 mg) were harvested and resuspended in 500 μL of phosphate-buffered saline (1 × PBS) containing 10 μM of the fluorescent probe supplied in the kit [29]. After incubation in the dark at 37 °C for 30 min, fluorescence intensity was measured using a microplate reader (Eppendorf, Hamburg, Germany) at an excitation wavelength of 510 nm and an emission wavelength of 610 nm. All experiments were performed in triplicate, and data were analyzed using GraphPad Prism 8.0.

### 2.8. Pathogenicity Assay

Pathogenicity of the *C. aenigma* and the Δ*Capex8* mutant was assessed on fresh strawberries and grapes using a needle wound inoculation method. Fruits were surface-sterilized with 75% ethanol, and a uniform wound (approximately 0.5 mm deep) was made at each inoculation site using a sterile needle. A 10 μL aliquot of a conidial suspension (10^6^ conidia/mL), harvested from 5-day-old cultures, was applied to each wound. We performed an assay using 10 strawberries and 10 grapes per strain. The inoculated fruits were incubated in the dark at 28 °C, and disease progression was monitored and recorded. At 5 days post-inoculation, photographs were taken, and the lesion areas were measured using ImageJ 1.51j8 software.

### 2.9. Fluorescence and Transmission Electron Microscopy (TEM)

For fluorescence microscopy, the subcellular localization of GFP and red fluorescent protein (RFP) fusions was detected on an Olympus FV3000 confocal microscope (Olympus Corporation, Tokyo, Japan). For TEM, collected mycelium blocks were processed by the institutional service platform, which performed chemical fixation, resin embedding, and ultrathin sectioning. Following this, the sections were examined using a Hitachi H7650 transmission electron microscope (Hitachi, Tokyo, Japan).

### 2.10. Quantitative Real-Time PCR (qPCR) Methods

For quantitative real-time PCR (qPCR), wild-type and Δ*Capex8* mutant strains were grown on CM plates for 4 days. Mycelia were collected, and total RNA was extracted from aerial mycelia using an RNA extraction kit (Shangya, Hangzhou, China) following the manufacturer’s instructions. RNA was reverse transcribed into cDNA using a kit (Tiangen, Beijing, China). The cDNA samples were directly used for qPCR. Reactions were performed on a real-time PCR instrument (Eppendorf, Hamburg, Germany) with ChamQ SYBR qPCR Master Mix (Vazyme, Nanjing, China) according to the manufacturer’s guidelines. Gene expression levels were calculated using the 2^−ΔΔCt^ method [30], with the wild-type sample as the calibrator. The *40S* ribosomal protein gene (XM_037328913.1) and *Actin* gene (XM_037319953.1) were used as internal controls. Duncan’s multiple range test was applied to evaluate significant differences among samples. The primers used in this study are listed in Table S1.

## 3. Results

### 3.1. Identification of CaPex8 in C. aenigma

**Figure 1:**
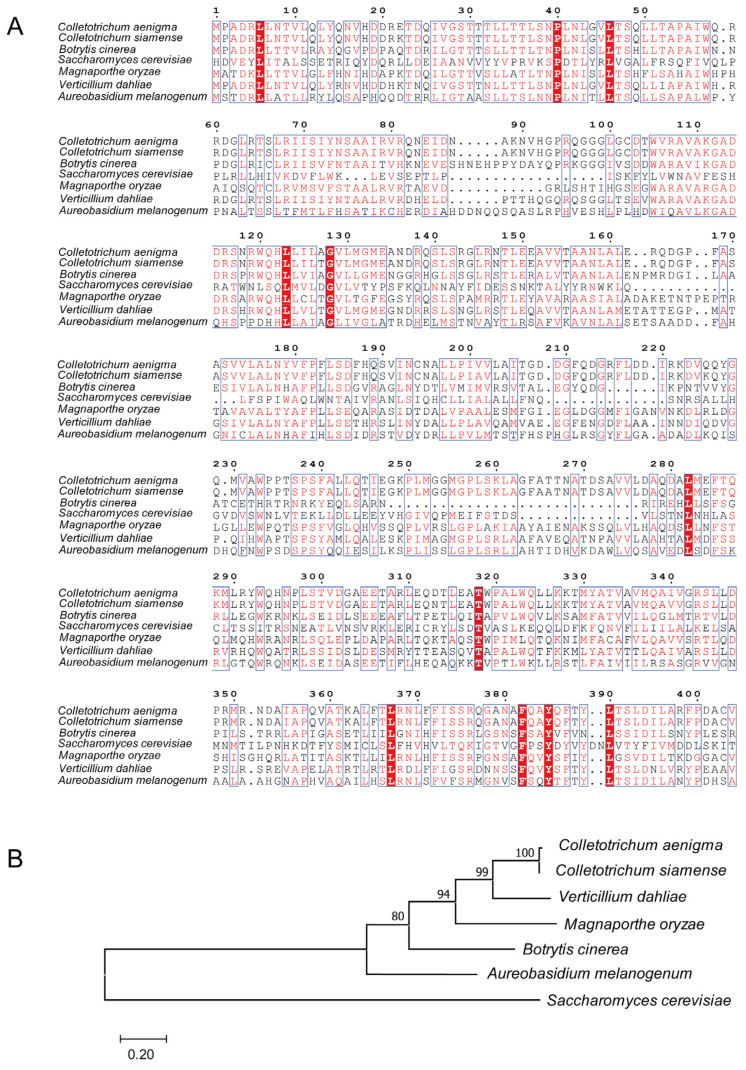
Identification of CaPex8 in *C. aenigma*. (**A**) A multiple sequence alignment was performed using MEGA 11.0 with the following Pex8 amino acid sequences from *Saccharomyces cerevisiae* (NP_011591.1), *Colletotrichum siamense* (KAI8269078.1), *Botrytis cinerea*(KAK6597450.1),*Candida albicans* (XP_711002.2), *Penicillium mononematosum* (XP_057145323.1), *Verticillium dahlia* (XP_009657113.1), and *Magnaporthe oryzae* (XP_003712892.1), Conserved amino acid residues are boxed in blue, and identical residues are highlighted in white on a red background. (**B**) Phylogenetic tree of the Pex8 protein from different filamentous fungi was constructed by the neighbour-joining method with MEGA 11.0.

Sequence analysis revealed that the CaPex8 protein consists of 682 amino acids. The full-length protein sequence was obtained from the NCBI database (XP_037178807.1). Homology analysis and sequence alignment showed that CaPex8 shared high amino acid identity with its ortholog from *Colletotrichum siamense* (98.63%), while exhibiting moderate to low identity with Pex8 from *Magnaporthe oryzae* (48.53%), *Botrytis cinerea* (45.81%), *Verticillium dahliae* (64.35%), and *Aureobasidium melanogenum* (38.56%). These results indicate that the amino acid sequence identity of Pex8 varies among different fungal species (Figure 1A). A phylogenetic tree was constructed using full-length Pex8 protein sequences from eight fungal species. This analysis revealed that *C. aenigma* Pex8 clusters more closely with orthologs from filamentous fungi *V. dahliae*, *C. siamense*, *M. oryzae*, *B. cinerea*, and *A. melanogenum* than with that from *S. cerevisiae* (Figure 1B).

### 3.2. CaPex8 Localizes to Peroxisomes

**Figure 2:**
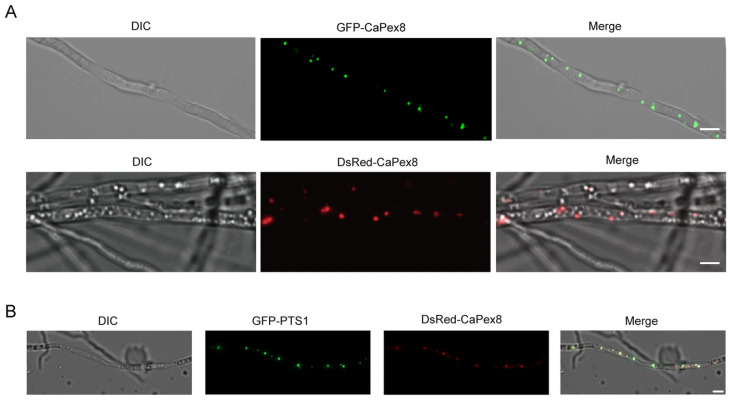
CaPex8 localizes to peroxisomes. (**A**) Localization of CaPex8 in *C. aenigma*. GFP-CaPex8 and DsRed-CaPex8 exhibit punctate distribution within hyphae. Scale bar, 5 μm. (**B**) Colocalization analysis of a hypha co-expressing GFP-PTS1 and DsRed-CaPex8. The punctate signal of DsRed-CaPex8 partially overlaps with that of GFP-PTS1, indicating peroxisomal localization. Scale bar, 5 μm.

To determine the subcellular localization of CaPex8, we performed colocalization assays using the peroxisomal marker GFP-PTS1. Fluorescence microscopy of hyphae co-expressing DsRed-CaPex8 and GFP-PTS1 showed that the DsRed signal clearly colocalized with the GFP-PTS1 puncta, confirming peroxisomal targeting of CaPex8 (Figure 2B). Consistently, hyphae expressing either GFP-CaPex8 or DsRed-CaPex8 exhibited a similar punctate fluorescence pattern (Figure 2A), indicating that the fluorescent tags did not interfere with its native localization. Collectively, these results demonstrate that CaPex8 localizes to peroxisomes.

### 3.3. Construction and Validation of the CaPEX8 Knockout Mutant

**Figure 3:**
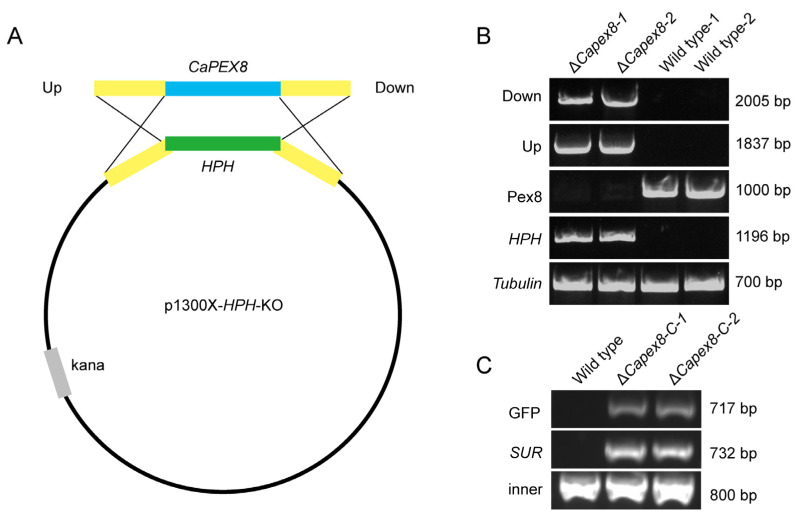
Construction and validation of the *CaPEX8* knockout mutant. (**A**) Strategy for *CaPEX8* disruption via homologous recombination with an *HPH* cassette. Diagram showing that the CaPex8 coding region was replaced by the *HPH* cassette. (**B**) Molecular verification of the Δ*Capex8* mutant. Validated mutants showed PCR products for the upstream and downstream recombination junctions and the *HPH* cassette, and PCR analysis using *CaPEX8*-specific primers (with *β-tubulin* as a control) confirmed the absence of the wild-type gene in the mutants. (**C**) Molecular verification of the complemented strain Δ*Capex8*-C. The validated complemented strain showed PCR products for the GFP tag and the *SUR* cassette, and PCR analysis using *CaPEX8*-specific primers confirmed that both Δ*Capex8*-C and the wild-type strain contained the expected product.

To explore the biological function of CaPex8 in the development of *C. aenigma*, we constructed a knockout vector carrying the hygromycin B resistance cassette (*HPH*). This vector was subsequently transformed into the wild-type strain (Figure 3A). Putative deletion mutants were screened for hygromycin resistance and verified by a series of PCR assays, including checks for gene replacement and genomic integrity (Figure 3B). A verified knockout strain (Δ*Capex8*) was thus obtained. To confirm that phenotypes observed in Δ*Capex8* were directly attributable to the loss of *CaPEX8*, we generated a complemented strain (Δ*Capex8*-C) by reintroducing the *CaPEX8* gene into the Δ*Capex8* mutant. The presence of GFP, the *SUR* selectable marker gene, and the Pex8 gene was confirmed by PCR (Figure 3C).

### 3.4. CaPex8 Is Essential for Peroxisomal PTS1 Matrix Protein Import

**Figure 4:**
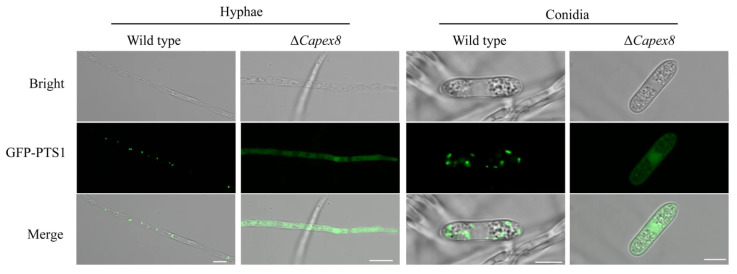
CaPex8 is essential for peroxisomal matrix protein import. Fluorescent localization of GFP-PTS1 in the wild-type and Δ*Capex8* mutant. In the wild-type, GFP-PTS1 manifests as punctate structures, whereas in the Δ*Capex8* mutant, GFP fluorescence is dispersed throughout the cytoplasm. Scale bar, 5 μm.

We assessed whether CaPex8 is involved in peroxisomal protein import by comparing the localization of GFP-PTS1 in wild-type and Δ*Capex8* strains. In the hyphae and conidia of the wild-type strain, GFP-PTS1 exhibited bright, stable, punctate fluorescence, confirming its proper targeting to peroxisomes. Conversely, the Δ*Capex8* mutant exhibited a complete mislocalization of GFP-PTS1 in both hyphae and conidia, which showed a diffuse distribution throughout the cytoplasm (Figure 4). This result suggests that the PTS1 protein failed to be imported into peroxisomes, indicating that CaPex8 is involved in the import of peroxisomal matrix proteins in *C. aenigma*.

### 3.5. CaPex8 Is Involved in Conidiation in C. aenigma

**Figure 5:**
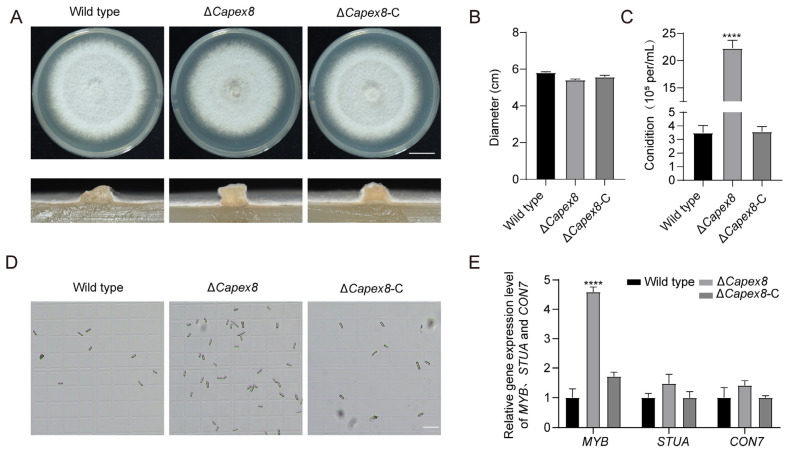
CaPex8 negatively regulates conidiation *in C. aenigma*. (**A**) Hyphal growth of the wild-type, the Δ*Capex8* mutant, and Δ*Capex8*-C strains. Scale bar, 1 cm. (**B**) Count and analyzed the colony growth diameter of wild-type, the Δ*Capex8* mutant, and Δ*Capex8*-C strains. Data analyzed using GraphPad Prism 8.0 and unpaired two-tailed Student’s *t*-test. (**C**) Statistical analysis of conidia production. Data analyzed using GraphPad Prism 8.0 and unpaired two-tailed Student’s *t*-tests. Asterisks indicate statistically significant differences (“****”: *p* < 0.0001). (**D**) Conidia of wild-type, the Δ*Capex8* mutant, and Δ*Capex8*-C strains. Scale bar, 50 μm. (**E**) The relative expression levels of *MYB*, *StuA*,and *CON7* in the wild-type and Δ*Capex8* mutant strains were determined by qPCR. Data analyzed using GraphPad Prism 8.0 and unpaired two-tailed Student’s *t*-tests (“****”: *p* < 0.0001). Error bars denote standard errors of three independent experiments.

Compared to wild-type and Δ*Capex8*-C, the Δ*Capex8* mutant showed no discernible effect on mycelial growth and aerial hyphal density after 4 days. (Figure 5A,B). Next, we examined the conidiation in the wild-type and Δ*Capex8*-C and Δ*Capex8* mutant. The Δ*Capex8* mutant displayed a striking increase in conidiation, producing approximately 7-fold more conidia than the wild-type strain [wild-type produced (3.0 ± 0.8) ×10^4^ conidia mL^−1^, while the Δ*Capex8* mutant produced (20.9 ± 2.1) ×10^4^ conidia ml^−1^] (Figure 5C,D). These results indicate that while CaPex8 is dispensable for vegetative growth, but essential for conidiation. qPCR analysis revealed a significant upregulation of *MYB*, a sporulation-related transcription factor gene, in the Δ*Capex8* mutant, while the expression of *CON7* and *StuA* remained unchanged. *MYB* family transcription factors are positive regulators of conidiation [31,32,33,34], whereas *CON7* is primarily involved in spore morphogenesis and also plays a role in influencing spore production [35,36], and *StuA* is critically required for normal conidiophore architecture and spore production [37]. Therefore, the upregulation of the *MYB* gene is likely one of the reasons for the enhanced sporulation capacity of the Δ*Capex8* mutant (Figure 5E).

### 3.6. CaPex8 Is Involved in Lipid Metabolism

**Figure 6:**
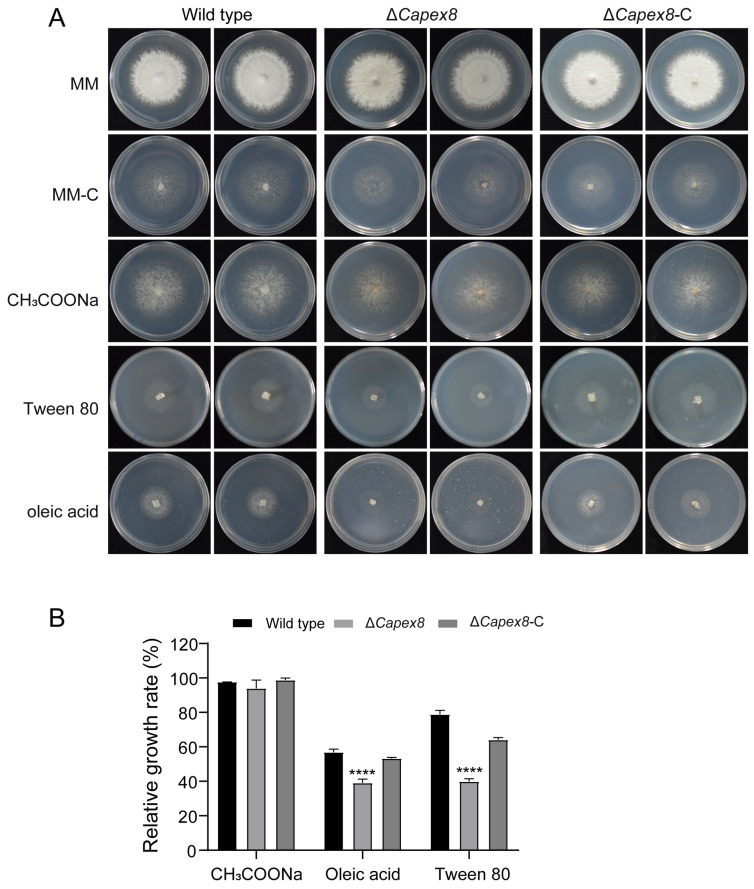
CaPex8 is involved in lipid metabolism. (**A**) Wild-type, the Δ*Capex8* mutant, and Δ*Capex8*-C strains were point-inoculated on MM and MM supplemented with the indicated carbon sources (0.1% oleic acid, 0.1% Tween 80, or 50 mM sodium acetate), followed by incubation at 28 °C for 7 days. (**B**) Relative growth rates of wild-type, the Δ*Capex8* mutant, and Δ*Capex8*-C strains. Statistical significance was assessed using *t*-tests (“****”: *p* < 0.0001). Error bars denote standard errors of three independent experiments.

Peroxisomes serve as the primary site for β-oxidation of long-chain fatty acids in cells, playing a critical role in lipid metabolism. We evaluated the growth of the Δ*Capex8* mutant on minimal medium (MM) containing various lipid-related carbon sources. Compared to the wild-type and Δ*Capex8*-C strains, the growth of the Δ*Capex8* mutant was severely impaired on MM supplemented with 0.1% oleic acid, 0.1% Tween 80 (a source of the long-chain fatty acid oleate), or 50 mM sodium acetate (a short-chain carbon source) (Figure 6A,B). When oleic acid was used as the carbon source, the average relative growth rate of the Δ*Capex8* mutant was 39.05%, representing a decrease of 17.74% compared to that of the wild-type strain (56.80%). When Tween 80 was used as the carbon source, the relative growth rate of the mutant decreased to 39.86%, which was 38.95% lower than that of the wild-type strain (78.81%). These results indicate that deletion of the *CaPEX8* gene impairs lipid metabolism in *C. aenigma*.

### 3.7. CaPex8 Is Essential for Multiple Stress Responses

**Figure 7:**
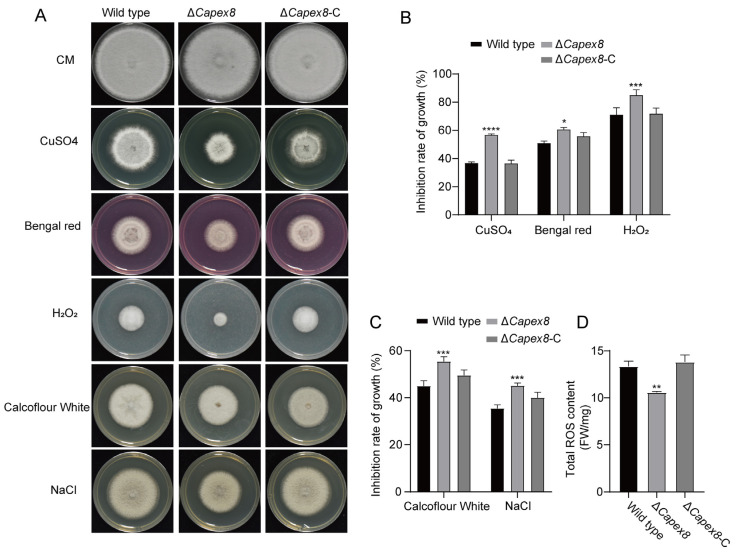
CaPex8 is essential for multiple stress responses. (**A**) Wild type, the Δ*Capex8* mutant, and the Δ*Capex8*-C strain on CM with 5 mM CuSO_4_, 50 μM Bengal Red, 0.1% H_2_O_2_, 50 μg/mL calcofluor white, and 1 M NaCl were cultured for 5 days. Each set of tests was repeated three times. (**B**,**C**) Quantitative analysis of growth inhibition under. (**B**) oxidative stress (5 mM CuSO_4_, 50 µM Bengal Red, 0.1% H_2_O_2_) and (**C**) cell wall or osmotic stress (50 µg/mL Calcofluor White, 1 M NaCl). (**D**) Quantitative analysis of total ROS levels based on red fluorescence intensity. The data were analyzed by GraphPad Prism 8.0 and an unpaired two-tailed *t*-test. Asterisks are used to indicate statistically significant differences. (“*”: *p* < 0.05; “**”: *p* < 0.01; “***”: *p* < 0.001; “****”: *p* < 0.0001). Error bars denote standard errors of three independent experiments.

To evaluate the impact of *CaPEX8* deletion on peroxisome-associated stress responses, we first assessed the reactive oxygen species (ROS) scavenging ability of the Δ*Capex8* mutant through sensitivity tests against various oxidative stress-inducing agents. As shown in Figure 7A,B, the Δ*Capex8* mutant exhibited strongly suppressed growth on complete medium (CM) supplemented with 0.1% H_2_O_2_, 50 μM Bengal Red, or 5 mM CuSO_4_, in contrast to the wild-type and Δ*Capex8*-C strains (Figure 7A,B). The antioxidant capacity of the Δ*Capex8* mutant was significantly impaired. Subsequent quantification of intracellular total reactive oxygen species (ROS) levels further demonstrated that the Δ*Capex8* mutant exhibited a notable reduction in ROS content relative to the wild-type strain (Figure 7D).

Given the links between peroxisomal function, cell wall integrity, and osmoadaptation, we further tested the mutant’s sensitivity to corresponding stressors. Growth of the Δ*Capex8* mutant was significantly reduced on CM supplemented with 50 µg/mL Calcofluor White (CFW) (Figure 7A,C), suggesting a defect in cell wall integrity. Similarly, under osmotic stress induced by 1 M NaCl, the colony diameter of the Δ*Capex8* mutant was significantly smaller than that of the control strains (Figure 7A,C), demonstrating enhanced sensitivity to osmotic pressure.

Collectively, these results indicate that CaPex8 contributes to ROS homeostasis, cell wall integrity, and osmotic adaptation in *C. aenigma*.

### 3.8. CaPex8 Is Required for Pathogenicity

**Figure 8:**
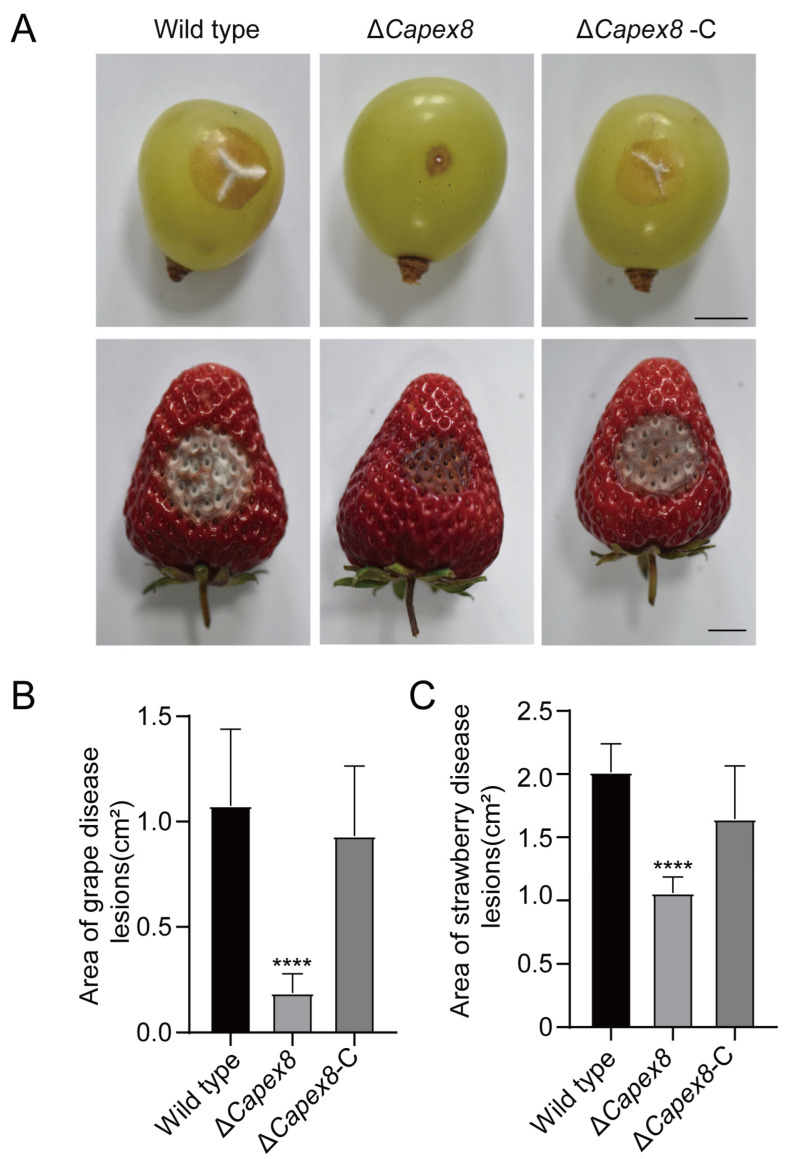
CaPex8 is required for pathogenicity. (**A**) Disease symptoms on wounded strawberries and grapes with conidial suspensions (10^6^ conidia/mL) of wild-type, Δ*Capex8*, and Δ*Capex8-C.* (**B**,**C**) Quantification of lesion areas on grape (**B**) and strawberry (**C**). The data were analyzed by GraphPad Prism 8.0 and unpaired two-tailed Student’s *t*-test. Statistically significant differences are shown as asterisks (“****”: *p* < 0.0001). Error bars denote standard errors of ten independent experiments.

To investigate the role of CaPex8 in the pathogenicity of *C. aenigma*, we performed infection assays on wounded strawberry and grape fruits. At the early stage of infection, the wild-type and Δ*Capex8*-C strains induced severe lesions, whereas the Δ*Capex8* mutant produced only limited, small, and faint lesions. Although the mutant eventually formed visible lesions around the inoculation site by 5 days post-inoculation, their expansion remained significantly restricted compared to the wild type and Δ*Capex8*-C (Figure 8A–C). These results suggest that CaPex8 contributes to pathogenicity in *C. aenigma*.

### 3.9. CaPex8 Is Essential for Peroxisome Biogenesis

**Figure 9:**
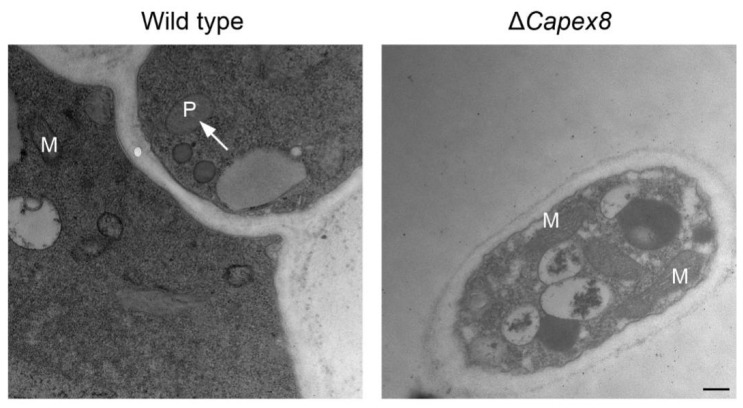
CaPex8 is essential for peroxisome biogenesis. Ultrastructure of wild-type and Δ*Capex8* mutants. Hyphae and conidia from 7-day CM plates were analyzed by TEM. Peroxisomes (P) and mitochondria (M) were detected in the wild-type, while the peroxisomes were absent in Δ*Capex8*. Scale bar, 0.2 μm.

To investigate the effects of *CaPEX8* deficiency on peroxisome structure, we compared the ultrastructure of the wild-type and the Δ*Capex8* mutant cells using transmission electron microscopy. In wild-type cells, spherical peroxisomes were primarily detected in the cell periphery and were distinguishable from mitochondria based on their shape and size. However, no peroxisomes or peroxisome-like structures were detected in the Δ*Capex8* mutant (Figure 9). These results indicate that CaPex8 is vital to peroxisome maintenance in *C. aenigma*.

## 4. Discussion

Peroxisome biogenesis is a well-established virulence determinant in plant-pathogenic fungi, including *M. oryzae* [38], *C. lagenarium* [39], and *F. graminearum* [19]. This process is orchestrated by conserved *PEX* genes, which encode proteins essential for matrix protein import, membrane assembly, and peroxisome proliferation [40]. Among these, *PEX8* serves as a critical peroxisomal matrix receptor and regulatory factor [10,20,41,42]. In this study, we identified and characterized CaPex8, the homolog of *S. cerevisiae* ScPex8 in *C. aenigma.* Phenotypic analyzes revealed that CaPex8 is required for peroxisome biogenesis, fatty acid utilization, cell wall integrity, and normal regulation of conidiation.

Consistent with its conserved role in peroxisomal matrix protein import [24,43,44], CaPex8-GFP fluorescence co-localized with the peroxisomal marker mCherry-PTS1, confirming its peroxisomal localization. Deletion of *CaPEX8* resulted in cytosolic mislocalization of the PTS1-tagged reporter and loss of visible peroxisomal structures under fluorescence microscopy. This confirms its essential role in the matrix protein import machinery. Previous studies have shown that Pex8 is a key component of the protein complex responsible for ubiquitination inside peroxisomes [44]; this process is essential for importing other proteins into the organelle. The import defect observed in the Δ*Capex8* mutant thus results from the loss of this conserved function. As a result of this import defect, the Δ*Capex8* mutant is impaired in lipid utilization, as shown by its reduced growth on media containing sodium acetate, Tween 80, or oleic acid as sole carbon sources, indicating impaired β-oxidation capacity.

This peroxisomal dysfunction leads to distinctive dysregulation of cellular redox homeostasis in the Δ*Capex8* mutant, which exhibits diminished basal ROS levels yet enhanced sensitivity to oxidative stress. This apparent paradox reflects the dual role of peroxisomes in both ROS generation and scavenging. Peroxisomes generate ROS primarily through fatty acid β-oxidation, where acyl-CoA oxidases produce H_2_O_2_ as a metabolic byproduct [20,43]. The impaired β-oxidation in Δ*Capex8* accounts for the decreased basal ROS production [20,45]. The peroxisome biogenesis defect in the Δ*Capex8* leads to the mislocalization of PTS1-tagged antioxidant enzymes, such as catalase, preventing their import into the peroxisomal matrix [43]. Thus, while reduced metabolic activity lowers the endogenous ROS level under basal conditions, the dysfunction of the peroxisomal antioxidant machinery renders the mutant incapable of counteracting exogenously induced oxidative challenge [46]. This redox imbalance, together with defective lipid metabolism, likely contributes to the compromised cell wall integrity and impaired osmotic adaptation observed in the Δ*Capex8* mutant. This conserved role of Pex8 in antioxidant defense is further supported by studies in *Candida albicans*, in which the Δ*pex8* mutant exhibits elevated sensitivity to miltefosine-induced ROS [23]. Interestingly, a similar phenomenon is observed in other fungal species beyond peroxisomal mutants. In *Trichothecium roseum*, the Δ*TrPLD1* and Δ*TrPLD2* mutants exhibit reduced ROS production under in vitro culture conditions, while showing increased sensitivity to oxidative stress (H_2_O_2_) and an impaired ability to scavenge exogenous ROS [47]. The decreased activities of catalase (CAT) and superoxide dismutase (SOD) in these mutants account for their susceptibility to exogenous H_2_O_2_. These results suggest that disruption of genes involved in lipid metabolism compromises redox homeostasis. In our study, the Δ*Capex8* mutant showed significantly inhibited growth in the presence of H_2_O_2_, Bengal Red, and CuSO_4_, further supporting the involvement of CaPex8 in oxidative stress response.

Peroxisomal proteins play critical roles in regulating key pathogenic processes, including fungal development, conidiation, and host infection [38,39,40,41]. The function of the *PEX* gene varies among different species, as illustrated by the functional divergence between CaPex8 in *C*. *aenigma* and its homolog in *B*. *cinerea*. Deletion of *BcPEX8* severely restricts both colony growth and conidiation, whereas the Δ*Capex8* mutant has no effect on hyphal growth but exhibits a marked increase in spore production. qPCR analysis revealed that this hyper-conidiation phenotype was accompanied by significant upregulation of a *MYB* gene, while expression levels of *CON7* and *StuA*—involved in spore morphogenesis and conidiophore architecture, respectively [35,36,37]—remained unchanged. *MYB* family transcription factors are well-established positive regulators of conidiation in filamentous fungi [33,34], and the significant upregulation of a *MYB* gene in *Aspergillus nidulans* has been shown to promote conidiation [31]. This suggests that CaPex8 may regulate conidiation in *C. aenigma* by modulating *MYB* expression, thereby driving the enhanced sporulation capacity of the Δ*Capex8* mutant. Further studies are required to fully elucidate the role of CaPex8 in conidiation.

In summary, this study demonstrates that the peroxisomal protein CaPex8 modulates fungal development and pathogenicity by regulating peroxisome biogenesis in *C. aenigma*. These findings indicate that CaPex8 plays an indispensable regulatory role in conidiation, peroxisomal function, and pathogenicity.

## Data Availability

The original contributions presented in the study are included in the article/Supplementary Material, further inquiries can be directed to the corresponding author.

## Supplementary Materials

The following supporting information can be downloaded at: https://www.mdpi.com/article/10.3390/jof12040241/s1, Table S1: Primers used in this study.
