# Supplementary material for: Characterization of CaPEX8 in Peroxisome Biogenesis and Pathogenicity of Colletotrichum aenigma
Source: J Fungi (Basel). 2026 Mar 26;12(4):241. doi: 10.3390/jof12040241 (PMC13118267; doi:10.3390/jof12040241)
Supplement: Supplementary file 1 [file jof-12-00241-s001.zip › jof-4137839-supplementary.pdf]

Table S1 Primers use in this study.

Primer name	Primer sequence (5'-3')
CaPex8up-F	AAAACGACGGCCAGTGCCAAGCTTCCCGGCGCGTGTCATTAAGGA
CaPex8up-R	CATTGATGTGTTGACCTCGGATCCCGTGAGCTGCAATCGCGACG
CaPex8down-F	CTTGACGAGTTCTTCTGAGGTACCGATGAAGAGGGTGGTAGTCG
CaPex8down -R	GACCATGATTACGAATTCGAGCTCTAATCGTTCTCCGCGCCAGG
CaPex8upyzF:	GATAGTTTAAACTGAAGGCGGG
CaPex8upyzR:	GACAAACGCACCAAGTTATCGT
CaPex8downyzF	ACCCATGGCGATGCCTGCTTG
CaPex8downyzR	CTCTCCCCGCGCGTTGGCCGA
CaPex8innerF:	ATGCCGGCGGACAGACTCCTCAA
CaPex8innerR:	CCCTTCATCCATTATCGCACCGC
HPH-F	TAGTGGAGGTCAACAATGAATG
HPH-R	CATCTACTCTATTCCTTTGCCC
TUB-1	AACATGCGTGAGATTGTAAGT
TUB-2	ACCCTCAGTGTAGTGACCCTTGGC
RED-CaPex8-F	ACCACCTGTTCCTGTCTAGAATGCCGGCGGACAGACTCCTCAACA
RED-CaPex8-R	TGTCGCTTACTGCAGGTCGACTAGTCTACTAGACCTCCCTTCATC
GFP-CaPex8-F	GACGAGCTGTACAAGTCTAGAATGCCGGCGGACAGACTCCTCAAC
GFP-CaPex8-R	TGTCGCTTACTGCAGGTCGACTAGTCTACTAGACCTCCCTTCATC
RTCamyb-F	TCCACGCAGTCTCACATCAC
RTCamyb-R	TGTCCCATCCTGATCTCGGT
RTCastuA-F	CTCGCATCAGGCATACTCGT
RTCastuA-R	TATCGTATGCACCGACCTGC
RTCa CON7-F	CAACAAGCAAGCAGTCCGTC
RTCa CON7-R	AAGGGTGCTGGTATGAGTGC
RTCa Actin-F	GGTGATGAGGCACAGTCGAA
RTCa Actin-R	AACCCTCGTAGATGGGGACA
RTCa 40s-F	AGTTCATCCGTAACCCCCTG
RTCa 40s-R	TTGTACAAGGAGCCGAGCTT
GFP-F	ATGGTGAGCAAGGGCGAGGAGCTG
GFP-R	CTTGTACAGCTCGTCCATGCCG
SUR-F	TTCCTGTCACCACCACTCTGCATG
SUR-R	CCATTGGGTCACCATTCCCTGC
Pex8inner-F800bp	ATGCCGGCGGACAGACTCCTCAAC
Pex8inner-R800bp	ATGGTCTGCAGCAGCGCAAATGAG
